# Traces across the body: influence of music-dance synchrony on the observation of dance

**DOI:** 10.3389/fnhum.2014.00965

**Published:** 2014-12-03

**Authors:** Matthew Harold Woolhouse, Rosemary Lai

**Affiliations:** ^1^Digital Music Lab, School of the Arts, McMaster UniversityHamilton, ON, Canada; ^2^McMaster Institute for Music and the Mind, McMaster UniversityHamilton, ON, Canada; ^3^Department of Psychology, Neuroscience and Behavior, McMaster UniversityHamilton, ON, Canada

**Keywords:** music and dance, synchronization, entrainment, eye movements, person perception

## Abstract

In previous studies investigating entrainment and person perception, synchronized movements were found to enhance memory for incidental person attributes. Although this effect is robust, including in dance, the process by which it is actuated are less well understood. In this study, two hypotheses are investigated: that enhanced memory for person attributes is the result of (1) increased gaze time between in-tempo dancers; and/or (2) greater attentional focus between in-tempo dancers. To explore these possible mechanisms in the context of observing dance, an eye-tracking study was conducted in which subjects watched videos of pairs of laterally positioned dancers; only one of the dancers was synchronized with the music, the other being asynchronous. The results were consistent with the first hypothesis—music-dance synchrony gives rise to increased visual inspection times. In addition, there was a preference for upper-body fixations over lower-body fixations across both synchronous and asynchronous conditions. A subsequent, single-dancer eye-tracking study investigated fixations across different body regions, including head, torso, legs and feet. Significantly greater dwell times were recorded for head than torso and legs; feet attracted significantly less dwell time than any other body region. Lastly, the study sought to identify dance gestures responsible for torso- and head-directed fixations. Specifically we asked whether there are features in dance that are specially designed to direct an observer’s gaze towards the face—the main “communicative portal” with respect to the transmission of intent, affect and empathy.

## Introduction

Watched by an average 11.2 million viewers each week—42.6% of the United Kingdom’s total viewing audience (Broadcasters’ Audience Research Board^1^)—the 2010 series of *Strictly Come Dancing* ranks as one of British broadcasting’s most successful (BBC, 2008). Although the program’s mass appeal was, and continues to be, due partly to the inclusion of famous individuals and the participatory feedback afforded by phone-in voting (Enli, 2009; Enli and Ihlebæk, 2011), it is questionable whether glamor and elimination-voting alone can account for either the show’s longevity or popularity. Nor is its appeal limited to the United Kingdom. *Dancing With The Stars*, based on the format of *Strictly Come Dancing*, had until recently spawned more international spin-offs than any other program in history (Allen, 2010), which begs the question, why is watching people dance so compelling?

In addition to *Strictly’s* reality-television appeal (Nabi et al., 2003), Wood (2010) proposes that “kinesthetic empathy” may be responsible for viewers’ motivation and commitment to dance programs. Kinesthetic empathy is based on the mirror mechanism, a neurobiological process whereby, in response to the observation of physical activity, we internally simulate movement sensations, speed, effort, and changing body configurations (Hagendoorn, 2004). Discovered in the brains of macaque monkeys circa 1990 (di Pellegrino et al., 1992; Gallese et al., 1996; Rizzolatti et al., 1996), mirror neurons in the parieto-frontal cortical circuit of the pre-motor cortex were found to become active during both action implementation and observation, thereby unifying action execution and action perception. Subsequently, the mirror mechanism was delineated in cortical areas of birds, monkeys other than macaques, and humans, and is now thought to underpin our internal, first-hand experience of the motor goals and intentions of others; see Rizzolatti and Sinigaglia (2010) for review. Thus dance programs, in which physical grace and motoric elegance are foregrounded, may represent a kind of virtual workout for the viewer in which, through the mirror mechanism, we are able vicariously to experience the poise and athleticism of dancers better trained than ourselves.

Moreover, evidence from dance and emotion research suggests that humans are highly attuned to dancers’ affective states, communicated through body and limb movements (see Bläsing et al., 2012, for overview). In a series of studies, researchers at the University of Jyväskylä in Finland have explored the ways and extent to which people are able to communicate the emotional content of music and their mental states through dance. Using motion capture, Luck et al. (2010) recorded the music-induced movements of a subgroup of people who had extreme scores in response to the Big-Five Inventory of personality (John et al., 2008). Principal-component analysis reduced movements to five main types—local movement, global movement, hand flux, head speed and hand distance—which were found to be associated in differing degrees with different personality attributes. For example, extroverts had high speeds for head and hand, whereas neurotics had reduced levels for all but one of the main movement types, local movement. Burger et al. (2013) studied the relationship between emotion in music and quasi-spontaneous movements made in response to it. Using correlation techniques, computationally extracted features of the movement data were linked with perceptually assessed emotional features of the music. Their analysis indicated that given a particular musical emotion, people move in characteristic ways. In experiments investigating the relative influence of vision vs. audition in evaluating musical performances, Tsay (2013) showed that visual information outweighs auditory, even when, as in the case of music, sound is believed to be preeminent. Stevens et al. (2009) studied the consistency of observers’ emotional reactions to dance using pARFs, portable personal-data devices designed to recorded continuous responses via hand-held styli. In their study exploring emotional arousal and valence, there was greater agreement in continuous ratings of arousal than valence; arousal appeared to be related to surface features of the performed work, such as acrobatic movement and intensity of the accompanying music.

Together, these and other emotion-based studies (e.g., Dittrich et al., 1996; Sawada et al., 2003; Saarikallio et al., 2013) indicate that personality traits can be expressed in distinct, quantifiable ways through dance, which, in combination with music’s emotional cues, can be read and interpreted by observers (Davidson, 2005). Dance, as well as stimulating mirror neurons in the pre-motor cortex, giving the viewer a visceral rather than merely intellectual experience, also elicits emotional responses, which can at times be richly empathetic (Berrol, 2006; Leigh, 2010; Reason and Reynolds, 2010).

Alongside apparent neurological and psychological benefits of watching dance, the rhythmic, spatiotemporal coordination of two or more individuals, otherwise referred to as “entrainment” (Phillips-Silver and Keller, 2012), may serve important socio-cultural functions. Three principal, socially based hypotheses, relating to courtship, coalition signaling and social bonding, have been advanced to account for the historical and geographical ubiquity of dance within human culture (Hanna, 1987). The courtship (or sexual-selection) hypothesis proposes that dance is a medium in which individuals can safely engage in peer-to-peer familiarization (Grammer et al., 1998; Miller, 2000; Luck et al., 2012a) and, with its emphasis on agility and coordination, mate-fitness selection (Brown et al., 2005; Luck et al., 2012b). The coalition-signaling hypothesis states that music and dance have evolved to allow groups of individuals to coordinate their actions for mutual benefit, such as reinforcing territorial boundaries or alliance building (Hagen and Hammerstein, 2009; Wiltermuth and Heath, 2009; Phillips-Silver et al., 2010). Hagen and Bryant (2003) support this hypothesis with evidence from chimpanzees, our closest extant non-human relatives (Lewontin, 1998), which employ territorial displays through chorused vocalizations and coordinated tree-buttress drumming (Arcadi et al., 1998). Lastly, social-bonding theories hold that dance and music co-evolved culturally and biologically as technologies for the promotion and maintenance of group cohesion, particularly beyond nuclear families (Cross, 2008; Cross and Woodruff, 2009; Phillips-Silver, 2009; Honing, 2013). And in this respect, human agency in the transmission of dance appears to be particularly potent. For example, the importance of observing human enactments of dance was demonstrated by Cross et al. (2009), who showed that novice dancers were able significantly to improve performance when tasked with learning novel dance steps in the presence of human models vs. abstract visual cues. Moreover, visual sensitivity to coordinated human action appears to be key to perceived group quality (Tsay, 2014), which is in turn related to social cooperation (Valdesolo et al., 2010). For foundational, theoretical work on music and social bonding, see Freeman (2000) and Merker (2000).

In this eye-tracking study, we investigate factors within dance (and music) that influence how people attend to dancers, and therefore that might contribute to dance as a social-bonding phenomenon. Our work builds on previous research by Woolhouse and Tidhar (2010), concerning entrainment and person perception, in which in-tempo dancing with others was found to enhance memory for incidental person attributes. Two interconnected hypotheses motivated Woolhouse and Tidhar’s study: first, coordinated action enhances inter-personal memory; and second, enhanced inter-personal memory is, in some way, associated with increased social affiliation, cooperation and bonding. The second hypothesis was not tested directly due to it being somewhat self-evident; how is it possible to bond with someone without remembering them? Woolhouse and Tidhar investigated the first hypothesis, that coordinated action enhances inter-personal memory in the context of group dancing.

Although not necessarily dealing with memory directly, findings of numerous studies support the notion that coordinated joint action increases cooperation (Hove and Risen, 2009; Kirschner and Tomasello, 2010; Rabinowitch et al., 2013; Cirelli et al., 2014), including dance (Behrends et al., 2012). Indeed, seemingly so strong is the link between joint action and cooperation, and far reaching its implications, Sebanz et al. (2006) concluded “[t]he ability to coordinate our actions with those of others is crucial for our success as individuals and as a species” (p. 70). With respect to the idea that coordinated action enhances interpersonal memory, earlier support for this was found by Macrae et al. (2008) who investigated the influence of hand waving between participants and experimenter on the ability of participants to recall words spoken by the experimenter and recognize her face in post-experiment memory tests. Results showed that in-phase hand waving, i.e., synchronous movement, caused more words to be recalled and greater recognition of the experimenter’s face. Macrae et al. suggested that synchronous hand-movements between experimenter and participant might have promoted mutual eye contact, which has been shown to expedite facial recognition and person categorization (Hood et al., 2003; Mason et al., 2004; Vuilleumier et al., 2005).

Woolhouse and Tidhar (2010) employed “silent-disco” technology in their study, which enabled them to transmit music surreptitiously at different tempi via radio headphones to subgroups of participants dancing within larger groups. Their method was briefly as follows. Pre-experiment the dancers’ faces were photographed; post-experiment each dancer was presented with the faces of the other dancers and asked to recall various memory targets, different colored sashes and symbols worn by each dancer. Results showed that in-tempo dancers remembered each other’s memory targets to a significantly greater degree than out-of-tempo dancers. A question that flows from this finding—and that we address here—is whether the enhanced memory effect was due to increased dwell times between in-tempo dancers, or whether some other effect, perhaps relating to attention, was responsible.

Two types of eye movements are typically studied in scene-perception research: fixations, during which the eyes remain relatively still and new information is acquired from the visual field, and saccades, movements between fixations during which vision is suppressed and little or no new information is gained (Rayner, 2009). In reading research, regressions—reverse saccades in which the eyes backtrack to the previous fixation point—are frequently examined in relation to syntactic comprehension (Liversedge and Findlay, 2000; Rayner et al., 2006). In scene perception, of a streetscape for example, fixations are usually 260–330 ms, interspersed with saccades lasting about 50 ms (Rayner, 2009). Saccade lengths can differ significantly depending on the type of image being viewed, and are about 40% longer for complex natural scenes than abstract patterns (Andrews and Coppola, 1999). Fixation durations, too, can differ for a variety of reasons, including attention and viewer expertise. Although attention may shift covertly within a single fixation (Duncan et al., 1994), durations are symptomatic of overt attention given to particular image regions; longer durations are associated with more complex material that require greater concentration and increased processing time (Rayner, 1998). Fixations of experts observing familiar material tend to be shorter than novices, which may be due to the application of task-specific schemata recalled from long-term memory, resulting in accelerated comprehension (Henderson, 2003). For example, trainee pilots fixate on flight-recording instruments almost twice as long as expert pilots (Bellenkes et al., 1997). In a study examining the influence of expertise on observing dance, Stevens et al. (2010) found that people with extensive dance training had significantly shorter fixation durations and faster saccades than novice dancers.

Expertise and experience, among other things, also influence not just how we look, but where we look. Expert radiologists have been found to target their attention on specific “high-risk” areas of radiographs, while novices scan radiographs evenly (Kundel and La Follette, 1972). And with respect to humans observing humans, although heterosexual men tend initially to fixate on women’s hip and/or breast regions (Dixson et al., 2011), in general, and certainly among infants, there is a preference for face-oriented gaze (Simion et al., 1998; Judd et al., 2009). Returning to Stevens et al. (2010), in an analysis of body-region viewing, the highly experienced choreographer in their study with veridical knowledge of the observed dance attended mostly to the head of the dancers, while novices attended more or less evenly to the head, neck, torso and arms. Stevens et al. proposed that the distinct viewing patterns of the experts in their study was “likely guided by the expectancies and schemata in long-term memory”, and that this was due in part to being “adept at abstracting and extracting key information from complex movement material” (p. 23). In sum, then, eye movements—principally fixations and saccades—are influenced by type of image being viewed and expertise, which, in turn, are indicative of viewers’ underlying cognitive processes and resources, attentional focus, and schematic knowledge.

As previously stated, we sought to determine whether the enhanced memory effect found by Woolhouse and Tidhar (2010) was due to increased dwell times between in-tempo dancers or perhaps some other eye-movement effect. In pursuit of this, we concentrated on three primary measures: dwell time (overall time spent gazing at a particular area of the screen), saccade length (measured as screen distance in mm), and fixation duration (mean fixation length in seconds for a particular area of the screen). In the group dances of Woolhouse and Tidhar’s study, individuals danced with in-tempo and out-of-tempo dancers, and therefore had the opportunity to observe people whose movements were either synchronous or asynchronous to their own and the musical beat. Our experiment was a lab-based equivalent of this, in which people’s eye movements were tracked. Here, participants, tapping along to music presented via headphones, simultaneously observed videos of two dancers, only one of which was synchronized to the beat of the music. If increased dwell time was responsible for enhanced inter-personal memory between in-tempo dancers, participants in the study should direct their gaze (and attention) predominantly towards the in-tempo, synchronous dancer and away from the asynchronous dancer. If, however, something other than overall time observing the synchronous dancer was responsible for the memory effect, such as attentional focus (Eriksen and Murphy, 1987), variables such as fixation durations or saccade distances might significantly differ depending on whether the synchronous or asynchronous dancer was being watched.

Finally, despite not being the focus of our study, our stimuli allowed us to investigate possible color biases with respect to observing dance. Although color-preference studies have been described as “bewildering, confused and contradictory” (McManus et al., 1981), some colors have been found to have relatively consistent connotations; for example, red is commonly associated with danger and mistakes, blue with peace and tranquility (Mehta and Zhu, 2009). We investigated whether dwell times, or some other aspects of eye movements, differed for dancers wearing blue vs. red clothing. Materials, methods and results of our study are now described.

## Materials and methods

### Participants

Twenty (11 males, nine females) university students between the ages of 18 and 24 (mean [*M*] = 19.9; standard deviation [*SD*] = 1.5) participated in the study. Of these, 18 were right handed, two were left handed, and all had normal or corrected-to-normal vision. Six participants had previously undertaken formal dance training (*M* = 3.8 years); 14 had received no dance training. In response to the question “Approximately how often do you dance?” two participants replied once a week, seven replied once a month, three replied once a year, and eight stated that they never dance. In respect of musical training, 14 participants had had formal training (*M* = 5.6 years); six participants had received no formal musical training. Across the twenty participants there was a slight, but not significant, negative correlation between number of years of formal musical training and formal dance training: *r*(18) = −0.06; N.S. Participants received one course credit for their involvement in the study and gave written informed consent in accordance with the requirements of the Ethics-Review Board of the host institution. Participants had no previous exposure to the test materials.

### Materials

Three popular music tracks from albums attaining top-twenty status in the United Kingdom Albums Chart (British Phonographic Industry, 2013) were selected for the study: “Unnatural Selection” by Muse (161 bpm), “Dimestore Diamond” by Gossip (93 bpm) and “The Water” by Hurts (73 bpm), which we categorized respectively as fast, medium and slow. The songs were chosen partly due to their danceability—each has a “catchy” rhythmic feel—and also because their tempo relationships are not simple integer ratios. If, for example, two tracks had had a two-to-one tempo relationship, the production of stimuli in which the music and dance were asynchronous would have been problematic—people might interpret someone dancing at half speed as still being “in time” with the music.

The visual element of the stimuli consisted of videos of a female dancer, recorded using a Sony HDV 1080i camera, free dancing against a white screen. The dancer was first filmed wearing a red outfit, dancing to the fast-, medium- and slow-paced pieces of music; then dancing to the same three pieces of music wearing a blue outfit. The dancer’s two outfits were stylistically matched. As mentioned above, the purpose of filming the same dancer wearing matching, differently colored outfits was to test for color bias with respect to observing dance. The videos were subsequently edited using FinalCut Pro X, which included size and brightness adjustments.

For the double-dancer study, pairs of medium- and slow-paced dance videos were combined using a 50:50 split screen, such that two laterally positioned dancers were presented simultaneously, i.e., side by side. The reason for choosing the medium and slow pieces was to explore the dwell-time hypothesis (described in Introduction) using two pieces with relatively close tempi, i.e., 93 bpm and 73 bpm. In the split-screen videos, only one dancer was synchronized with the music, either medium or slow, the other was asynchronous. That is, in each trial one dancer danced in time to either medium or slow music, while the other dancer danced out of time. In addition, by removing the audio from the videos altogether, silent, control stimuli were created with the intention of examining how dance in the presence of music might affect eye movements. Finally, in order to control for visual lateral bias (Guo et al., 2009), two sets of split-screen videos were produced in which the side-by-side positions of the dancers were switched: red outfit on the left and blue on the right, blue outfit on the left and red on the right. In combination with the music-dance factor, these manipulations produced 12 videos in total; see Table 1. Each video lasted 60 s.

**Table 1:** **Split-screen video stimuli used in the double-dancer study**.

		Dancer on left			Dancer on right		
Video	Music	Dancer	Outfit color	Dancer synchronized?	Dancer	Outfit color	Dancer synchronized?
1	Medium	Medium	Red	Yes	Slow	Blue	No
2	Medium	Medium	Blue	Yes	Slow	Red	No
3	Medium	Slow	Red	No	Medium	Blue	Yes
4	Medium	Slow	Blue	No	Medium	Red	Yes
5	Slow	Medium	Red	No	Slow	Blue	Yes
6	Slow	Medium	Blue	No	Slow	Red	Yes
7	Slow	Slow	Red	Yes	Medium	Blue	No
8	Slow	Slow	Blue	Yes	Medium	Red	No
9	Silent	Medium	Red	NA	Slow	Blue	NA
10	Silent	Medium	Blue	NA	Slow	Red	NA
11	Silent	Slow	Red	NA	Medium	Blue	NA
12	Silent	Slow	Blue	NA	Medium	Red	NA

*The “Dancer synchronized?” column indicates whether the tempo of the dancer matched the tempo of the music*.

For the single-dancer study the color factor was disregarded; consequently only the videos of the dancer wearing the blue outfit were presented. The dancer was centrally positioned in the videos, danced to either the fast or slow tempo, i.e., at either 161 bpm or 73 bpm, and was either synchronous or asynchronous to the music. As with the double-dancer stimuli, silent stimuli were created by removing the music from the videos. These manipulations produced six videos in total; see Table 2. Each video lasted 60 s.

**Table 2:** **Video stimuli used in the single-dancer study**.

Video	Music	Dancer	Dancer synchronized?
1	Fast	Fast	Yes
2	Fast	Slow	No
3	Slow	Fast	No
4	Slow	Slow	Yes
5	Silent	Fast	NA
6	Silent	Slow	NA

The visual component of the stimuli (either double- or single-dancer videos) was presented on a LG 27” desktop monitor with a screen resolution of 1920 × 1080. The music was presented through AKG K 172 HD headphones, which have a frequency range of 18 Hz–26 kHz. Each participant adjusted the headphone volume to a comfortable level prior to the first trial of the experiment session.

### Procedure

All participants completed a questionnaire containing their personal information, and levels of dance and musical experience, including years of formal training. Participants were briefed on the general functioning of the eye-tracking system and seated in a booth, approximately two feet in front of the monitor and eye-tracking camera. The apparatus was calibrated and validated using a nine-point grid. Participants were informed as to how the stimuli would be presented and instructed to look at the monitor throughout the experiment. In order to maintain attention on the music, as well as the visual element of the dancing, participants were instructed to tap on a computer keyboard spacebar positioned in front of them in time to the musical beat. The experiment consisted of two trial blocks—the double-dancer block followed by the single-dancer block. Each participant was presented with the videos in a different random order within each trial block. To avoid fatigue, participants were invited to take a short break between the blocks. The experiment lasted approximately 30 min.

### Eye-tracking camera

Eye movements were recorded using the Mirametrix S2 Eye Tracker at a sampling rate of 60 Hz for both eyes, although only data recorded from the right eye were used in the subsequent analyses. The bright-pupil tracking system (sometimes referred to as “red eye effect”, caused by on-camera-axis illumination; see Holmqvist et al., 2011, for detailed summary) has a 0.5-degree accuracy range, drift rating of <0.3 degrees, and allows users to move their heads within the width-height-depth range of 25 × 11 × 30 cm. The eye-tracker equipment sits unobtrusively below the monitor, facing the user. Data on all participants were exported with the system’s EyeMetrix Software (Mirametrix Inc.).

### Data analysis

For the double-dancer, each stimulus was divided into two laterally positioned regions of interest (ROI) of equal dimensions, covering the left and right dancer. For each ROI the proportion of dwell times, saccade lengths, and fixation durations were analyzed. Each dependent variable was entered as means per participant per video, apart from dwell times, which were entered as gross totals per participant per video.

For the single-dancer, each stimulus was divided into four ROIs, corresponding to the head, torso, legs and feet of the dancer. For each ROI the proportion of dwell time was calculated for each participant per video. In addition to the ROIs, we investigated whether particular dance gestures created consistent patterns of fixations between participants, and whether at certain moments the dancer moved in such a way as to draw observers’ attentions to particular parts of her body. Accordingly, a moving average of fixation positions was calculated by advancing a 500 ms time window through the fixation data at 250 ms intervals. Subsequently, overall fixation centroids were calculated by taking an average of all fixation positions per 500 ms time window, sufficient to allow for saccade latency, i.e., the 175–200 ms required to encode the position of a new visual target and initiate an eye movement (Becker and Jürgens, 1979). Similarly, an overall fixation-position SD per time window was calculated by taking the mean of the SD of the fixations’ x-axis coordinates and the SD of their respective y-axis coordinates. The fixation centroid per time window and associated SDs enabled the locus of the fixations to be tracked throughout each video, and also the dance gestures responsible for producing more tightly clustered, consistent fixation patterns. An advantage of this relatively simple approach was that it allowed the effect of all three manipulations—synchronous music and dance, asynchronous music and dance, silent dancing—to be projected onto a single video simultaneously; see supplementary materials.

### Statistical analysis

For the double dancer, repeat-measure three-way analyses of variance (ANOVA), with *Music-Dance*, *Color* and *Lateral Position* as within-subject factors, were run separately on dependent variables dwell time, saccade length and fixation duration. Within *Music-Dance* there were six levels: (1) medium music, medium dance; (2) slow music, slow dance; (3) medium music, slow dance; (4) slow music, medium dance; (5) no music, medium dance; and (6) no music, slow dance. Thus (1) and (2) represented levels in which the music and dancer were synchronized; levels (3) and (4) where the music and dancer were asynchronous; and (5) and (6) silent dancing. The two levels within *Color* were the red and blue outfits of the dancer, and the two levels within *Lateral Position* were left and right.

For the single dancer, a repeat-measure one-way ANOVA with *Body* as within-subject factor was run on dependent variable dwell time. There were four levels within factor *Body*, the ROIs associated with the head, torso, legs and feet of the dancer. Our analysis also explored a possible association between overall fixation location with respect to the dancer’s body and fixation dispersion. Consequently, Pearson product moment correlation coefficients were calculated between fixation centroid and fixation-position SD per 500 ms time window for the fast- and slow-dance videos.

Tukey’s Honest Statistical Difference (HSD; Miller, 1981) was used for multiple comparisons in factors with more than two levels, i.e., *Music-Dance* and *Body*. All data were analyzed using the open-source statistical package *R* (2.15.0, GUI 1.51). MATLAB (R2013a) was used to associate the videos of the dancer with fixation centroids and SDs per time window. In order to reduce “jitter” and “flicker” effects of the eye-tracking system and possible artifacts of its data-parsing algorithm, fixations below 100 ms were omitted from the analysis; for discussion on the relative merits of omitting fixation durations below a certain threshold and data-processing algorithms, see Wass et al. (2013).

## Results

### Double dancer

#### Dwell time

**Figure 1:**
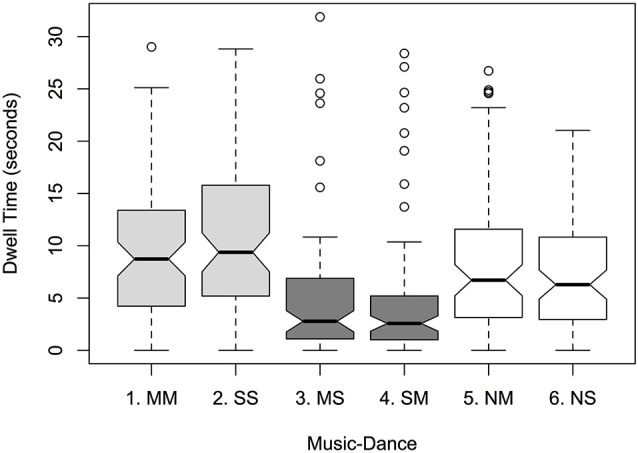
**Notched boxplot of mean dwell times per *Music-Dance* level: MM (medium music-medium dance); SS (slow music-slow dance); MS (medium music-slow dance); SM (slow music-medium dance); NM (no music-medium dance); NS (no music-slow dance)**. Synchronous levels are indicated in light gray, asynchronous in dark gray, and no-music levels in white. Notches extend to +/−1.58 IQR/sqrt(n). If two boxes’ notches do not overlap, there is “strong evidence” that their medians differ at roughly a 95% confidence interval (Chambers et al., 1983; p. 62).

Within *Music-Dance*, a greater proportion of dwell time was found for synchronous levels over silent, and for silent over asynchronous: 21.1% and 23.9% for medium music-medium dance (MM) and slow music-slow dance (SS) respectively; 18.6% and 15.6% for no music-medium dance (NM) and no music-slow dance (NS); 10.7% and 10.2% for medium music-slow dance (MS) and slow music-medium dance (SM); see Figure 1. This was supported by a significant main effect of *Music-Dance* on dwell time (*F*_5,456_ = 11.651; *p* < 0.001). There was no significant effect of *Color* on dwell time (*F*_1,456_ = 0.645, ns), nor of *Lateral Position* (*F*_1,456_ = 0.714, ns). Pairwise comparisons of levels within *Music-Dance* (using Tukey’s HSD procedure) yielded patterns of significance based largely on whether the levels presented synchronous or asynchronous music with respect to the dancer, or were silent. For example, comparison of synchronous pairs MM-SS, asynchronous pairs MS-SM, and silent pairs NM-NS were not significant (*p* = 0.832, 0.999 and 0.796 respectively). Whereas, comparisons between synchronous and asynchronous pairs MM-MS, MM-SM, SS-MS and SS-SM were highly significant (*p* < 0.001). Comparisons between synchronous and silent pairs, and asynchronous and silent pairs yielded a mixed picture of significant and non-significant differences. There were no significant interactions between factors.

#### Saccade length

**Figure 2:**
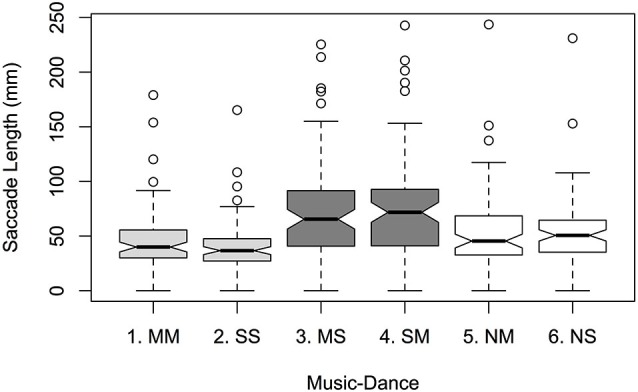
**Mean saccade lengths per *Music-Dance* level**.

Within *Music-Dance*, greater mean saccade lengths were observed for asynchronous levels than synchronous and silent: 71.11 mm and 72.51 mm for MS and SM respectively; 54.34 mm and 51.83 mm for NM and NS; 45.99 mm and 41.26 mm for MM and SS; see Figure 2. This was supported by a significant main effect of *Music-Dance* on saccade length (*F*_5,456_ = 9.372; *p* < 0.001). As with dwell time, there was no significant effect of *Color* on saccade length (*F*_1,456_ = 0.492, ns), nor of *Lateral Position* (*F*_1,456_ = 0.018, ns). Pairwise comparisons within *Music-Dance* also yielded a similar pattern of result to that of dwell time: synchronous pair MM-SS, asynchronous pair MS-SM, and silent pair NM-NS did not differ non-significantly (*p* = 0.969, 0.999 and 0.998 respectively). All synchronous-asynchronous pairwise comparisons, such as MM-MS and SS-MS, were significant (*p* < 0.001), as were all asynchronous-silent pairings (except MS-NM which was marginally significant; *p* < 0.1). All synchronous-silent pairs yielded non-significant differences. There were no significant interactions between factors.

#### Fixation duration

Mean fixation durations were somewhat similar within *Music-Dance*: 0.159 s and 0.161 s for MM and SS respectively; 0.154 s and 0.164 s for MS and SM; 0.163 s and 0.156 s for NM and NS. Given the closeness of the means, unsurprisingly there was no significant main effect of *Music-Dance* on fixation duration (*F*_5,456_ = 0.494; ns). Likewise, there was no effect of either *Color* on fixation duration (*F*_1,456_ = 0.101, ns), nor of *Lateral Position* (*F*_1,456_ = 0.001, ns).

### Single dancer

#### Dwell time

**Figure 3:**
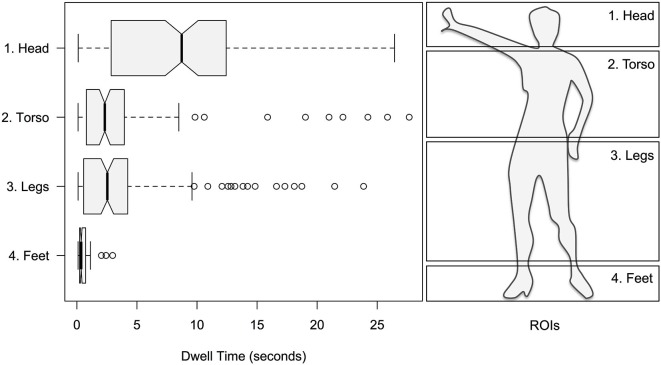
**Left panel**: mean dwell times for the head, torso, legs and feet of the dancer. **Right panel**: corresponding ROIs.

As stated previously, the focus of the single-dancer study was to explore eye-movements with respect to different parts of the dancer’s body. Within factor *Body* a greater proportion of dwell time was found for head over torso and legs, and torso and legs over feet: 50.51% for head; 22.37% for torso; 24.29% for legs; 2.83% for feet; see Figure 3. This was supported by a significant main effect of *Body* on dwell time (*F*_3,476_ = 56.19; *p* < 0.001). All pairwise comparisons of levels within *Body* were highly significant (*p* < 0.001), except for levels torso and legs, which did not significantly differ.

**Figure 4:**
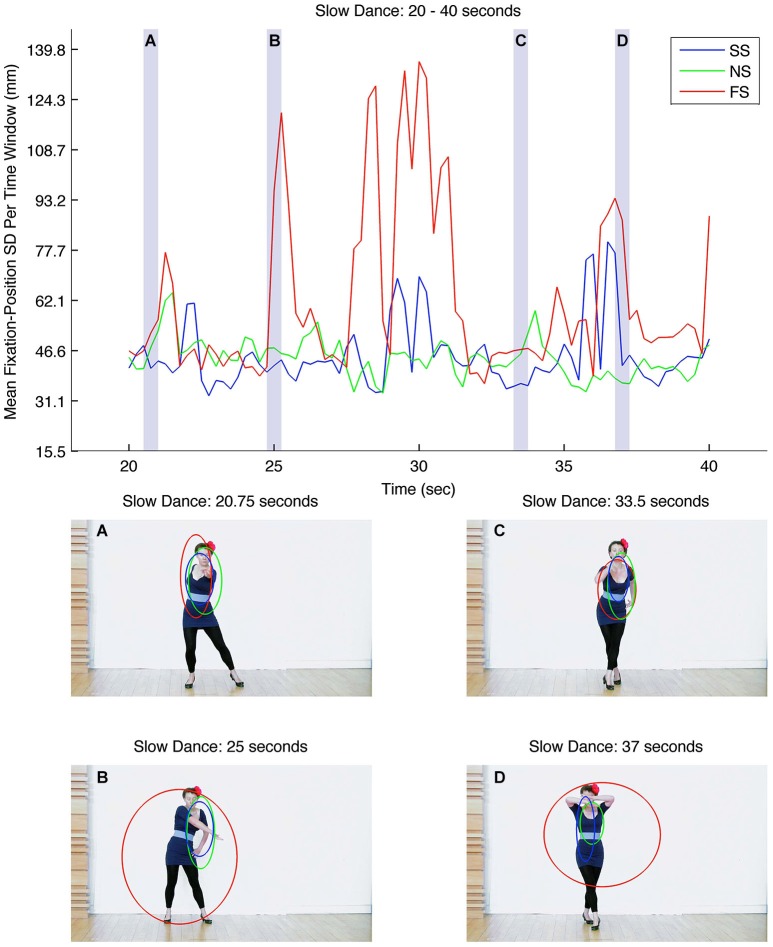
**Top**: graph showing mean fixation-position SDs for each 500 ms time window for the middle 20-s of the slow-dance video (seconds 20–40). **Bottom**: Still-frames **(A)**, **(B)**, **(C)** and **(D)** correspond to the transparent, light-gray vertical bars in the graph above. The blue, green and red ellipses correspond to levels SS, NS and FS, and show the relative sizes of the mean fixation-position SDs and fixation locations at each point in the video.

As previously described, a moving-average procedure was carried out in order to explore the extent to which certain dance gestures created consistent patterns of fixations. The top panel in Figure 4 shows the mean fixation-position SD for each 500 ms time window for the middle 20 s of the slow-dance video (seconds 20–40). The three lines represent slow music-slow dance (SS; blue line), fast music-slow dance (FS; red line), and no music-slow dance (NS; green line). Three level types are therefore present within the figure: synchronous music and dance (SS); asynchronous (FS); and silent (NS). Immediately apparent are the relatively large fluctuations in mean fixation-position SDs for FS in comparison to SS and NS; that is, at certain moments in the asynchronous level, observers’ fixations are very widely distributed across the dancer’s body, whereas at other moments their fixations are more tightly clustered. This is in sharp contrast to the effects of the synchronous and silent levels on fixation-position SDs, which stay within a lower, narrower band.

**Figure 5:**
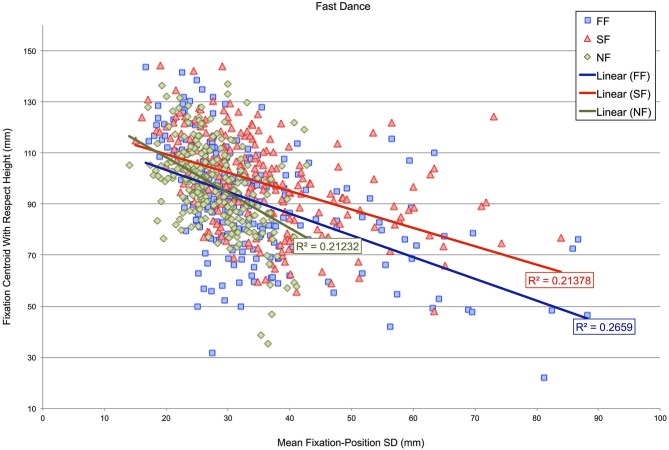
**Scatterplot of fixation-centroid height against mean fixation-position SD per 500 ms time window for videos in which the dancing is fast**. Three levels are present within the plot: fast music-fast dance (FF), slow music-fast dance (SF) and no music-fast dance (NF).

**Figure 6:**
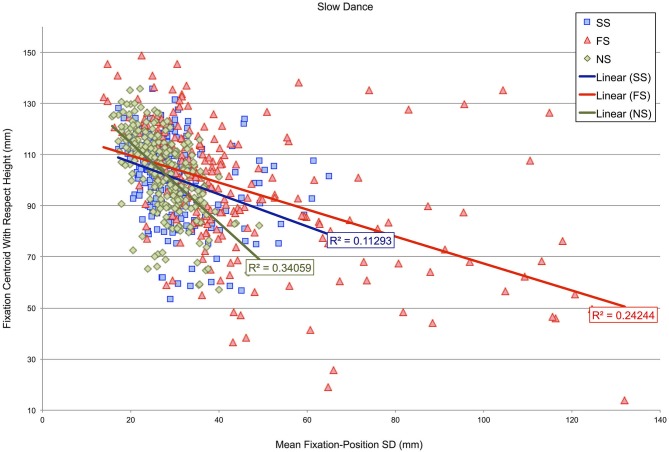
**Scatterplot of fixation-centroid height against mean fixation-position SD per 500 ms time window for videos in which the dancing is slow**. Three levels are present within the plot: slow music-slow dance (SS), fast music-slow dance (FS) and no music-slow dance (NS).

Subsequently, an analysis determined whether the height of fixation centroids with respect to the dancer’s body might correspond with fixation-position SDs in either the fast- or slow-dance videos. Figures 5, 6 show scatterplots of fixation centroids with respect to height against mean fixation-position SD per 500 ms time window for the fast- and slow-dance videos. Three levels are present within each plot: fast music-fast dance (FF), slow music-fast dance (SF) and no music-fast dance (NF) in Figure 5; SS, FS and NS in Figure 6. The two plots therefore show the overall fixation height and fixation-position dispersion of each 500 ms time window for each type of level: synchronous, asynchronous and silent. Also shown are the linear regression lines and r-square values of each level. Although none of the r-square values are especially high (the highest, NS, is 0.34059), the regression lines show that there is a consistent negative correlation between height on the dancer’s body and fixation-position SD; which is to say, in general, that when fixation centroids were located on the head, fixations were more compact, i.e., observations were more similarly located, than when fixation centroids were located on progressively lower parts of the body. This observation is supported by the Pearson’s product moment correlation coefficients associated with the plots’ r-square values, all of which were significant at *p* < 0.001: FF, *r*(238) = −0.516; SF, *r*(238) = −0.477; NF, *r*(238) = −0.461; SS, *r*(238) = −0.336; FS, *r*(238) = −0.477; NS, *r*(238) = −0.584.

## Discussion

A core finding of this study is that when presented with two dancers simultaneously, only one of whom is synchronized with the music, participants overwhelmingly spend more time gazing at the synchronous dancer than asynchronous. Demonstrated in pairwise comparisons of levels within *Music-Dance* in the double-dancer experiment, the effect was observed irrespective of the tempo of the music. For example, MM and MS levels differed significantly as did SS and SM levels. This point was reinforced by the non-significant differences between synchronous pair MM-SS and asynchronous pair MS-SM. In addition, removing the music altogether created no preference for either fast dancing or slow; i.e., dwell times for silent pair NM-NS did not differ significantly. Clearly, dwell times were influenced by the presence of music and whether it was synchronous or asynchronous with the dancing.

The finding lends support to the conjecture that the enhanced-memory effect found by Woolhouse and Tidhar (2010) was due to increased gaze times between in-tempo dancers, which may have led them to remember each other’s memory targets (sash colors and symbols) more than out-of-tempo dancers. Furthermore, the effect—if generalized to other types of synchronous-asynchronous behavior—could also help to explain the enhanced-memory results of Macrae et al. (2008), who showed that synchronous hand-waving caused people to recall more words and to remember the experimenter’s face with greater acuity. It would appear to be the case, as Macrae at al. suggested, that synchronous movement does indeed promote mutual eye contact, and with it increased person perception. It does not follow, of course, that this necessarily leads to increased social bonding, as Woolhouse and Tidhar suggested; however, it is plausible to hypothesize that this process may be an actuating mechanism through which social bonding—facilitated by increased memory for one another—could occur.

While overall dwell times look to be responsible for increased interpersonal memory between people engaged in synchronous action, fixation durations—a measure associated with attention (Rayner, 1998)—seem not to contribute to the effect. That is, there was no evidence in our study that people’s attention levels, as indicated by fixation durations, fundamentally altered between observing either synchronous or asynchronous stimuli, i.e., in-tempo or out-of-tempo dancers. And therefore no evidence that the enhanced-memory found by Woolhouse and Tidhar, and Macrae at al. was due to changes in attentional focus. Expertise was not measured in our study and therefore we were unable to corroborate the finding of Stevens et al. (2010), that highly trained dancers observing dance have shorter fixation durations than novices. We did, however, find an effect of saccade length, indicating that participants had exaggerated scan paths when observing out-of-tempo dancers. Thus, one effect of asynchronous music and dance is that observers’ fixations, in an apparent attempt to integrate perceptually disparate sensory inputs, scan relatively distant locations on the dancer’s body, seemingly seeking out points at which music and dancer align temporally. Whether or not this is indicative of a change in attention is presently unknown, and a question that we would like to pursue is whether the scan paths for desynchronized music and dance exhibit greater overall variability, and whether this indicates a change in cognitive load and/or processes by which successive fixation positions are selected.

Factors *Color*, a preference for dancers wearing blue or red clothing, and *Lateral Position*, a bias for dancers on the left or right, proved not to be significant with respect to any dependent variable. Although colors can be connoted to more-or-less specific phenomena (red and danger, for example), if color biases do exist with respect to dancers’ clothing, they appear to be overwritten by dancing itself, and, likewise, possible lateral biases. That being said, from what is known of the perceptual influence of color, the effects of dancers’ clothing tint and hue on qualities such as attractiveness, empathy, trust and willingness to cooperate clearly deserve further investigation. For example, the finding that exposure to the color green prior to executing “unusual-uses” tasks (Guilford, 1967) enhances creativity (Lichtenfeld et al., 2012) may indicate that dancers wearing viridescent costumes are attended to differently, perhaps with greater inquisitiveness by observers. Black and red clothing has been shown to effect within- and between-sex attractiveness ratings (Roberts et al., 2010). Our research did not test this; however, given that the stimuli consisted of the same dancer wearing matching red or blue outfits, the study could in principle have investigated possible interactions between music-dance synchrony and color in respect of attraction. Red’s redolent nature has also been shown to influence cognitive-performance tasks. Elliot et al. (2011) observed degraded performance on IQ tests amongst participants who briefly viewed red before the tests. In their study, color also had a physiological effect: concomitant with worse IQ performances, subjects exposed to red exhibited a decrease in high-frequency heart rate variability (often referred to as respiratory sinus arrhythmia). Whether matters of the heart are also responsible for the phenomenon of male customers giving higher tips to waitresses wearing red (Guéguen and Jacob, 2014) remains to be established. However, if, for example, an understanding of the role of color in dance costumes from a cross-cultural perspective is to be gained, at a minimum research will have to address the cognitive and physiological effects referred to above.

In the single-dancer study, perhaps paradoxically, people spent least time fixating on feet, arguably a dancer’s greatest asset (Macaulay, 2009). Similarly, relatively small dwell times were recorded for the main part of the dancer’s body (torso and legs) compared to her head; see Figure 3. Interestingly, a similar head-centric finding was also observed by Stevens et al. (2010) in the fixations of an expert choreographer, while novices’ initial fixations tended to be more evenly spread. However, despite this difference, Stevens noticed that aspects of novices’ eye movements began to resemble those of the expert on only the second viewing of the dance, suggesting the presence of a strong learning effect. Given that our videos were repeated within each trial block, it is likely that our participants’ eye movements may have become similar to those of an expert viewer, particularly with respect to body location, and hence head-based fixations predominate. Moreover, we speculate that the animated and exaggerated limb movements commonly associated with dance (Brown and Dissanayake, 2009), coupled with high movement sensitivity in peripheral vision (McKee and Nakayama, 1984), may mean that observers of dance do not need to attend directly to a dancer’s physical extremities, instead leaving them free to concentrate (and fixate) on the face, a main communicative portal with respect to the transmission of intent (Trevarthen, 1979), affect (Ekman and Friesen, 1971) and empathy (Schulte-Rüther et al., 2007).

The importance of head fixations in our study is also illustrated in Figures 5, 6. Here, mean fixation-position SDs per 500 ms time window are plotted against vertical height with respect to the dancer’s body. The significant negative correlations for both fast and slow videos, irrespective of the synchronous, asynchronous or silent manipulations, indicate that there was less variability in fixation locations when the fixation centroid moved towards the dancer’s head. While this is partly an effect of the predominant amount of time participants spent gazing at the head, which occupies a small area relative to the rest of the body, it may also have been the case that the dancer moved in such a way as to draw participants’ eyes specifically towards her head and face at certain moments, thereby creating lower fixation-position SDs for this region.

We undertook to identify some of the dance gestures that might give rise to head-centered fixations and gestures that may lead observers to fixate elsewhere. The four video still-frames at the bottom of Figure 4 correspond to the transparent, light-gray vertical bars in the graph above; blue, red and green ellipses represent the mean fixation-position SDs and fixation locations of the different levels at these points in the video. Small ellipses indicate that observers’ fixations were similarly located; conversely, large ellipses indicate that fixations were dispersed over a relatively large area. The still-frames were selected for illustrative purposes (rather than for rigorous methodological reasons) and show the gesture of the dancer at particular moments in time: A, 20.75 s; B, 25.0 s; C, 35.5 s; D, 37.0 s. In still-frame A the dancer points and looks directly at the camera; the ellipses of all three levels are small, overlapping and upper-torso/head focused, indicating similar patterns of observations within each level. In still-frame B the dancer looks away from the camera and points down with her right arm; in contrast to A the fixations associated with SS and NS closely and consistently follow her right arm, whereas those associated with the asynchronous level, FS, become highly diffuse (indicated by the large red ellipse). Still-frame C once again shows the dancer looking at the camera, this time leaning forward; here the dancer’s upper torso becomes the relatively narrow focus of attention in all three levels. Finally, still-frame D shows the dancer covering her face with her hands; as in B, levels SS and NS remain relatively narrow, although there is increased spread in the y-axis in level SS (indicated by the tallness of the blue ellipse). As in B, the fixation positions of the asynchronous level, FS, become highly diffuse.

Although the above frame analysis is selective, it suggests—at least in these instances—that participants met the dancer’s stare when it was directed towards the camera, i.e., at them, and looked elsewhere when the dancer averted her gaze, particularly in the asynchronous level, FS. Evidence supporting human sensitivity to gaze direction has shown that straight gaze direction has a special status, which the visual system processes more quickly and with greater accuracy than averted gaze (von Grünau and Anston, 1995). Future research could take a systematic approach to the issue of gaze direction and dance by seeking to ascertain how dance gestures and shared attention help to direct observers’ eye movements to particular parts of dancers’ bodies.

As with any stimuli based on a restricted set of exemplars, our study was limited by using only one dancer. While the dancer was highly skilled, and danced freely in a contemporary style typical of many people today, factors specific to this dancer not controlled for in the experiment may have skewed our results. As Luck et al. (2010) showed, dancing reveals aspects of a dancers’ personality, and therefore may or may not engender empathy in an observer. The movements of our dancer, however professionally executed, could have created particular emotional and arousal responses in our participants, leading them to observe her differently from someone else. Future research must establish the extent to which our case-based findings can be generalized, notably with respect to possible interactions between the sex of observers and dancers (see, for example, Luck et al., 2012b). A further limitation of the study is the question of whether data obtained from passively observing dance can be extrapolated to explain data obtained from active engagement in dance. Earlier in this discussion, we propose that our findings support the conjecture of Woolhouse and Tidhar (2010) that their enhanced-memory finding was due to increased gaze times between in-tempo dancers. While our proposal is plausible given our results, it is questionable whether an eye-tacking study, done by participants sitting at a computer monitor, fully captures the dynamism of people dancing in groups. Requiring participants to tap in time to the music, as we did, will have undoubtedly created active physical entrainment, but until a study involving a head-mounted mobile eye-tracking system is employed in the context of group dancing this question remains open. Moreover, memory with respect to synchronous and asynchronous movement was not tested directly. Therefore, while suggesting that eye movements may account for memory effects observed in previous studies, we acknowledge that research investigating the existence of a firm causal link between the two has yet to be undertaken.

A significant aspect of our research has been to show how the combination of dance and music, two temporal art forms, significantly impact eye movements, revealing time-dependent cognitive processes relating to the integration of multisensory information and attention. We have identified a possible mechanism—increased gaze time—by which synchronous action may lead to increased person perception, interpersonal memory and, by extension, social bonding. More broadly, understanding the possible implications of this will undoubtedly involve exploiting technological advancements, in mobile eye-tracking devices for example, and through incorporating theories and findings from other rapidly expanding areas of entrainment research.

## Conflict of interest statement

The authors declare that the research was conducted in the absence of any commercial or financial relationships that could be construed as a potential conflict of interest.

## Footnotes

^1^Available online at: http://www.barb.co.uk/. Accessed on May 1, 2014.
